# Effect of Guarana (*Paullinia cupana*) on Cognitive Performance: A Systematic Review and Meta-Analysis

**DOI:** 10.3390/nu15020434

**Published:** 2023-01-14

**Authors:** Brian Hack, Eduardo Macedo Penna, Tyler Talik, Rohan Chandrashekhar, Mindy Millard-Stafford

**Affiliations:** 1 School of Biological Sciences, Georgia Institute of Technology, Atlanta, GA 30318, USA; 2 Physical Education Faculty, Federal University of Pará, Castanhal 68746-630, PA, Brazil

**Keywords:** caffeine, cognition, attention, memory, nutraceuticals

## Abstract

The plant extract guarana is known for its caffeine content and other bioactive ingredients, which purportedly may improve cognitive performance. Recent reviews have examined the effects of chronic supplementation of guarana in clinical populations; however, the acute effects of guarana on cognitive tasks, while of interest, have produced mixed results. Whether acute guarana ingestion improves human cognitive performance was assessed by performing a systematic review coupled with a meta-analysis. Eight placebo-controlled studies were identified and met the inclusion criteria providing data on 328 participants. The dose of guarana (37.5 to 500 mg) with reported caffeine content (4.3 to 100 mg) varied. Effect sizes (ESs) were calculated as the standardized mean difference and meta-analyses were completed using a random-effects model. The ESs for guarana averaged across a variety of cognitive measures and outcome variables were less than trivial (Hedge’s g = 0.076, *p* = 0.14). Using a subgroup meta-analysis (Q = 12.9, *p* < 0.001), ESs indicating a faster response time for guarana vs. a placebo (g = 0.202, *p* = 0.005) differed from the accuracy measures (g = −0.077, *p* = 0.4) which were non-significant. For response time, guarana ingested in a capsule (g = 0.111) tended to differ (Q = 2.96, *p* = 0.085) compared to guarana when dissolved in liquid (g = 0.281). Meta-regression of the study ESs of overall cognitive task performance was not related to the guarana dose (R^2^ < 0.001) or to the time allowed prior to cognitive testing (R^2^ < 0.001). Acute guarana ingestion had a small effect on the response time (faster performance) during a variety of cognitive tasks without affecting the accuracy. Whether the changes were linked to the caffeine content or other bioavailable substances in guarana is unknown. Additional studies that directly compare matched doses of caffeine versus guarana are needed to understand its effects on cognitive performance.

## 1. Introduction

Nutritional ergogenic aids may increase human performance by different mechanisms. Some ingested substances may exert an influence on the central nervous system and/or by altering energy metabolism [1]. Improvements in performance may also be observed in different domains (physical, tactical, motor, cognitive, etc.). Such is the case for caffeine, the most widely consumed ergogenic aid worldwide. For example, approximately 85% of adults maintain a regular caffeine consumption in the United States and these numbers are in line with countries in Europe and the Americas [2]. Despite variation in population-based habitual consumption, moderate long-term caffeine intake (up to 400 mg/day) is not linked to adverse effects on male fertility, behavior, cancer risk, calcium balance, or cardiovascular health [3].

Although the most common source of caffeine consumption is black coffee, there are other natural sources. The natural plant extract of guarana *(Paullinia cupana*) is one popular example. Guarana, a native species from the Amazon region in South America, has been used for its stimulant and medicinal properties for centuries by indigenous communities of the region [4]. Pure guarana composition can contain up to 5.3% of caffeine [5], although typical caffeine sources such as espresso coffee [6] and dark chocolate [7] contain much less (about 0.21% and 0.08% of caffeine, respectively). Moreover, the chemical composition of guarana, which in addition to a relatively high concentration and bioavailability of methylxanthines, also contains flavonoids (e.g., catechins, epicatechins) and pro-anthocyanidins, which may have other potential positive impacts on human health [5]. These include a range of health outcomes associated with reductions in chronic fatigue in cancer patients [8,9], short-term weight and fat loss [10], and potentially protective effects against metabolic disorders in elderly subjects in habitual consumers [11]. The anti-inflammatory, antioxidant, anticancer, and hypocholesterolemic effects of guarana have also been demonstrated and reviewed previously [5]. However, a recent meta-analysis established that chronic supplementation of guarana did not reduce perceived fatigue in cancer patients and the quality of experimental evidence was rated as low [12].

The most consistent data regarding human performance and guarana ingestion has been focused on brain function, presumably because guarana is recognized for its psychoactive properties, especially given its caffeine content. Caffeine crosses the blood–brain barrier and acts upon the central nervous system through adenosine antagonism [13] which is associated with reduced perceived fatigue and improved alertness, vigilance, attention, and reaction time [14]. The pharmacokinetics of caffeine suggest that for most individuals, caffeine levels peak in the blood within 60 min following ingestion with a half-life of 3–5 h [15]. Therefore, the caffeine content in the guarana supplement utilized is a key research design factor. One of the first studies [16] examined the effect of acute guarana ingestion on memory and attention but found no difference compared to a placebo. However, in a later study [17], the acute ingestion of guarana improved mood and affective domains along with secondary memory [18]. Since then, additional studies have focused on cognitive performance effects due to guarana, some suggesting benefits [19,20,21], while others finding no effect [22,23]. Therefore, the effect of guarana ingestion on cognitive performance remains unclear.

There is an increasing interest in guarana ingestion focusing on human health and performance, evidenced by it being one of the more popular herbal nutritional supplements searched for by the general population [24]. Furthermore, since the ergogenic substances present in guarana likely have an action on the central nervous system [25,26], there is a high plausibility of identifying benefits in cognitive performance related to its consumption. Whether this acute benefit occurs consistently across various cognitive tasks should be examined. Therefore, our objective was to systematically review the published literature and conduct a meta-analysis to quantify the acute effects of guarana on task performance related to cognitive functions.

## 2. Materials and Methods

### 2.1. Systematic Review

A systematic review was conducted in order to identify studies pertaining to whether an acute dose of guarana influences cognitive performance. Here, an acute dose was defined as a one-time oral administration of guarana. The cognitive tasks considered were those that contained at least one dependent variable with an accuracy score or response time. The databases searched in this review were Medline, ProQuest Theses and Dissertations Global, PsychInfo, and PubMed. The databases were searched using the following search terms: (guarana OR “paullinia cupana”) AND (cogniti* OR memory OR inhibition OR “executive function” OR “response time” OR accuracy). An additional hand search was conducted for unpublished data. The search was performed on 13 September 2022.

### 2.2. Study Inclusion

Studies were reviewed for the following inclusion criteria: (1) examined an acute dose of guarana, (2) outcome measures reported data from a cognitive task, (3) written in English, and (4) the study was conducted on humans. Studies that included cognitive tasks both before and after exercise were retained, but data were extracted only for tasks that were administered prior to exercise. Studies not performed on healthy adults and evaluating cognitive results more than 24 h after ingestion were excluded. The research design of studies (e.g., randomization and familiarization) influencing study quality was not considered for study inclusion.

### 2.3. Study Selection

**Figure 1:**
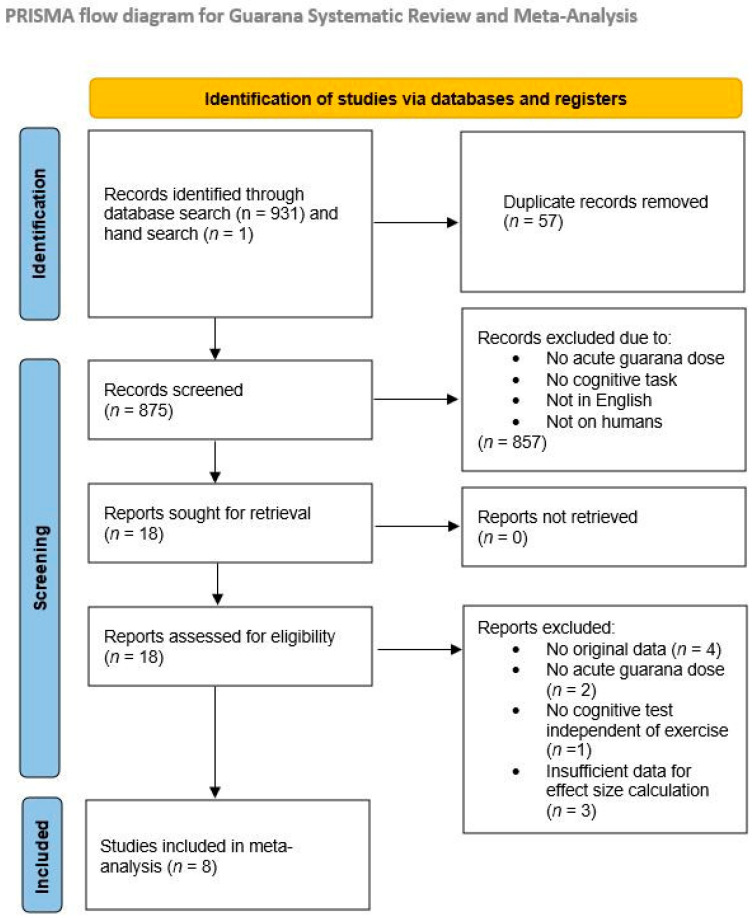
PRISMA flowchart detailing study identification.

A total of 931 records were returned. One additional unpublished study was included from the authors [22]. After the removal of duplicates (*n* = 57), the remaining 874 records were reviewed for inclusion by title and abstract by two separate reviewers using the criteria described above. In cases of disagreement, a third reviewer was consulted for a final decision. Studies that were not conducted on humans, did not report a guarana dose prior to cognitive testing, or not published in English were omitted from our analysis (Figure 1). After further evaluating the abstracts by the inclusion criteria, 18 studies were selected for a full-text review by two independent reviewers. Studies with no original data (*n* = 4) or no acute guarana dose (*n* = 2) were excluded. Twelve studies were initially selected for inclusion in the systematic review. Of these 12, one additional study [27] was excluded because all of the data were collected during exercise and guarana was not ingested (mouth rinse). Two more studies were excluded due to insufficient reported data to calculate the effect size [23,28]. One study [20] was excluded because the data were only reported for significant results, thus potentially inflating the overall study effect size on all of the cognitive tasks. Therefore, the total number of studies included for the meta-analysis was eight. This process used the preferred reporting items for systematic reviews and meta-analysis (PRISMA Figure 1) [29].

### 2.4. Data Extraction and Quality Assessment

For each selected article, data were extracted for all of the cognitive tasks. For each task, the mean, standard deviation, and sample size were collected for both the placebo/control supplement (might have contained other ingredients but matched to the guarana intervention) and guarana. Other interventions such as caffeine or carbohydrates may have also been included for comparisons (see Table S1). Correlational data for subject performance between the treatment groups were extracted where available but they were not reported in many studies. Each cognitive outcome extracted was designated as either a measure of accuracy or response time based on the provided units. For three studies [20,23,28], sufficient original data were not included in the paper, and despite attempts to contact the authors, data were unavailable. One additional study [27] included only cognitive tasks completed during exercise and was excluded. For the remaining eight studies, a quality assessment was conducted using the Physiotherapy Evidence Database (PEDro) scale [30]. The scale yields a total possible score of 11 points, with a greater score corresponding to higher quality.

### 2.5. Meta-Analysis

The meta-Analysis was conducted using comprehensive meta-analysis (CMA) software (Version 3; Biostat Inc., Englewood, NJ, USA). The mean, standard deviation, and sample size values were input for each cognitive task for each study that was available. Correlational data were input where available but they were not reported in several studies. Where a correlation was not reported, an estimate was made based on the correlations that were available. Calculated correlations from the available data sets ranged from 0.6 to 0.77, which appeared to be within the range of other cognitive task test–retest correlations surveyed [31,32]. For each cognitive task, a standardized mean difference was computed.

The effect sizes (ESs) were reported using Hedges’ g (g) due to the small sample size of several included studies to combat the bias found in Cohen’s d for small studies [33]. The ESs favoring guarana were entered as positive, while the ESs favoring the placebo were negative. Therefore, higher values reported for accuracy were considered favorable while a lower response time (faster) was also considered favorable. For studies with multiple dependent ESs, an overall study ES was calculated using the mean of all of the accuracy and response time outcome ESs. Despite the presence of multiple ESs from individual studies, multivariate meta-analysis was not practical in this instance due to the lack of availability of exact correlational data for many tasks.

Due to the wide variability in study designs present, a random effects model was employed. ESs were considered trivial from 0.1 to 0.2, small from 0.2 to 0.5, moderate from 0.5 to 0.8, and large above 0.8. Heterogeneity was assessed using a Q test, *I*^2^ (between-study) and *T*^2^ (within-study) values.

Duval and Tweedie’s trim and fill funnel plots were used to assess publication bias (i.e., tendency for significant findings to be published with higher priority than non-significant findings), which may result in a biased overall ES. These plots showed the study ES compared to the study standard error. Where publication bias is not present, the plot should be symmetrical around the overall ES. In cases of asymmetry, computed study suggestions show a change in ES if a sample study is added to reduce the publication bias.

### 2.6. Moderator Variables

Due to the variety in cognitive tasks and the different cognitive outcome variables (e.g., response time, percent accuracy, and change scores), and experimental conditions (guarana dose and time of test following guarana ingestion) observed across the studies included in the analysis, the role of moderator variables in explaining inter-study variation observed in the ESs was probed.

Each cognitive outcome was designated as a response time or an accuracy measure. A subgroup meta-analysis was used to investigate whether the ES from these outcome measures differed from each other by using a Q test statistic. Another subgroup analysis was used to investigate whether ESs for response time differed based on the ingestion vehicle (i.e., capsule or dissolved in liquid). For continuous variables (guarana dose and time following ingestion), a method-of-moments meta-regression was used to assess the relationship with ES. The dose was the mass of guarana (mg) ingested, not the caffeine dose within the guarana. The time that the cognitive testing was performed following guarana ingestion was also regressed relative to the ES. For each meta-regression, a cluster plot was created along with a correlation coefficient. For studies with more than one level of a variable (e.g., more than one dose), those levels were separated and treated as independent studies.

## 3. Results

### 3.1. Study Characteristics

Eight studies were included in the meta-analysis, providing data from a total of 328 participants, 142 of which were females (Table S1). Data from a total of 272 dependent variables were extracted from the cognitive tasks available. Among the included studies, six were repeated-measure crossover designs and the remaining two [16,19] were independent group designs. Sample sizes ranged from 10 to 130 participants with a median participant number of 27. Two studies [16,22] did not find any difference between treatment the groups, while the remaining six studies reported at least one significant difference in an outcome measuring cognitive performance. All eight studies had at least one accuracy measure and seven had at least one response time measure. Four studies used a capsule to administer the guarana, while the remaining four studies dissolved guarana powder in liquid. The guarana doses administered ranged from 37.5 to 500 mg with a median dose of 222.2 mg. The time of testing after guarana ingestion ranged from 15 to 360 min with a median time of 60 min. Seven studies had the maximum PEDro score of 11 while one study [34] had a score of seven due to participant dropouts and being single-blind (Table S2). All studies that reported participant age utilized adults with a mean age > 30 years. The earliest published study was from 1994.

### 3.2. Overall Effect of Guarana on Cognitive Task Performance

**Figure 2:**
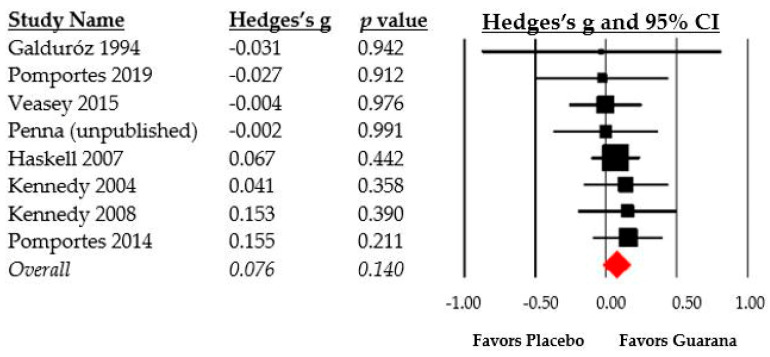
Forest plot of effect sizes from eight studies averaged across all outcomes (multiple doses, accuracy, and response time for several cognitive tests). Squares represent the effects size and its size proportional to the study weighting in the meta-analysis. Horizontal lines indicate 95% confidence intervals. The red diamond width indicates a 95% CI for the overall effect size [16,17,18,19,21,22,34,35].

Figure 2 presents a forest plot on the overall effect of acute guarana ingestion on cognitive task outcome measures. The combined ES averaged across all cognitive variables (test type and outcome measure) for guarana compared to the placebo was not significant, although it tended to favor guarana positively (g = 0.076, *p* = 0.140, 95% CI: −0.025 to 0.176). When the study by Scholey [20] was included (which provided data on only the two significant test results out of six total tests), the ES for guarana would have otherwise been trivial, but significant (g = 0.121, *p* = 0.041, 95% CI: 0.005 to 0.237). Exclusion of the unpublished study from Penna [22] did not alter the overall significance (g = 0.082, *p* = 0.124, 95% CI: −0.022 to 0.187) for the aggregated cognitive performance. Heterogeneity appeared to be low (Q = 1.576, *p* = 0.980). The between-study variation (*I*^2^ = 0%) and within-study variation (*T*^2^ = 0) were low. In total, studies ranged from an ES of −0.031 to 0.153. Four studies (50%) indicated a positive ES while four studies (50%) indicated a negative ES. A trim and fill analysis did not indicate that any suggested imputed studies were needed to minimize publication bias.

### 3.3. Moderator Variable Analysis

#### 3.3.1. Outcome Type

**Figure 3:**
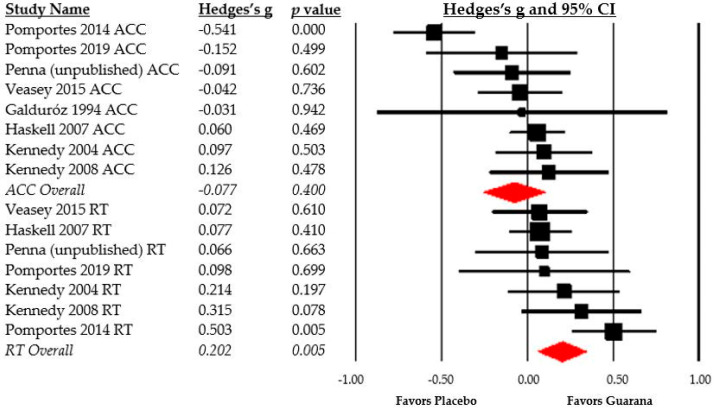
Forest plot of effect sizes for subgroup meta-analysis comparing accuracy (top) and response time (bottom). The red diamond represents the effect size and the CI for each group. ACC = accuracy and RT = response time [16,17,18,19,21,22,34,35].

Forest plots for outcome variables related to accuracy and response time are presented in Figure 3. The studies (*n* = 8) ranged from an ES of −0.541 to 0.126. Five studies had a negative ES for accuracy, while three studies indicated a positive ES. guarana did not result in a significant positive effect on accuracy (g = −0.077, *p* = 0.400, 95% CI: −0.258 to 0.103). Heterogeneity was significant (Q = 20.244, *p* = 0.007) with between-study variance moderate (*I*^2^ = 65.4%) and within-study variance low (*T*^2^ = 0.040). Trim and fill analysis indicated one suggested imputed study would minimize publication bias, with a small negative ES (~g = −0.4). The addition of a study with this ES would move the overall effect slightly more negative, but not alter the non-significant result.

Seven studies included at least one response time outcome for a cognitive task, and all of them indicated a positive ES. The studies ranged from an ES of 0.072 to 0.503. guarana resulted in a small positive effect size indicating a faster response time (g = 0.202, *p* = 0.005, 95% CI: 0.062 to 0.343). Heterogeneity was not significant (Q = 9.309, *p* = 0.157), with the estimated between-study variance moderate (*I*^2^ = 35.6%) and the within-study variance low (*T*^2^ = 0.012). Trim and fill analysis did not indicate any additional imputed studies were necessary to account for publication bias.

According to the subgroup analysis, the effect of guarana on response time was greater than the effect on accuracy (Q = 12.852, *p* < 0.001). With the removal of the unpublished study [22], there was little impact on the accuracy ES (g = −0.075, *p* = 0.473, 95% CI: −0.116 to 0.035) and response time (g = 0.216, *p* = 0.007, 95%: 0.093 to 0.315). The effect size for response time remained greater than the effect size for accuracy (Q = 12.488, *p* < 0.001). Due to one study [35] contributing both the lowest accuracy ES and the highest response time ES, an additional sensitivity analysis was performed by removing this study. When removing this study from the analysis, the ES for response time and accuracy did not differ from each other (Q = 1.504, *p* = 0.220). The ES for accuracy became more favorable towards guarana, but remained not significant (g = 0.024, *p* = 0.648). The ES for response time was lower, but remained significant (g = 0.124, *p* = 0.041).

#### 3.3.2. Ingestion Vehicle

**Figure 4:**
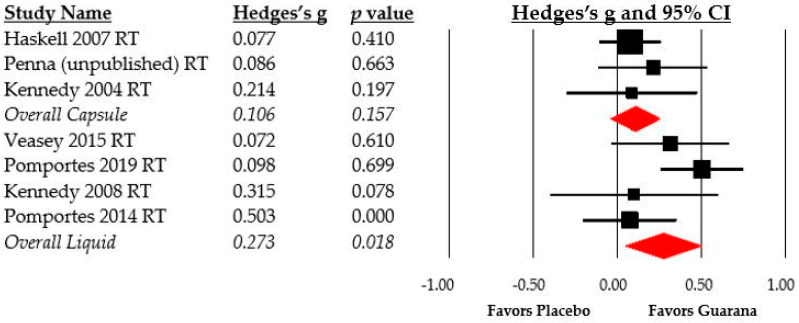
Forest plot of effect sizes for subgroup meta-analysis comparing guarana ingested either in the form of a capsule (top) or dissolved in liquid (bottom). The red diamond represents the effect size and the CI for each group [16,17,18,19,21,22,34,35].

Figure 4 presents the subgroup meta-analysis comparing guarana ingestion (capsule vs. dissolved in liquid) in response time measures of the cognitive tasks. There was no difference in the ES for guarana when ingested as a capsule compared to dissolved in liquid (Q = 2.961, *p* = 0.085). The ES for guarana when ingested in a capsule (m = three studies) did not affect the response time (g = 0.106, *p* = 0.157, 95% CI: −0.041 to 0.253). Heterogeneity was not significant (Q = 0.53, *p* = 0.767). However, the ES for guarana when dissolved in a drink (m = four studies) was small but significant for the response time (g = 0.273, *p* = 0.018, 95% CI: 0.047 to 0.499). Heterogeneity was not significant (Q = 5.818, *p* = 0.121). The estimated between-study variance was moderate (*I*^2^ = 48.4%) and the within-study variance was low (*T*^2^ = 0.025).

#### 3.3.3. Meta-Regression

**Figure 5:**
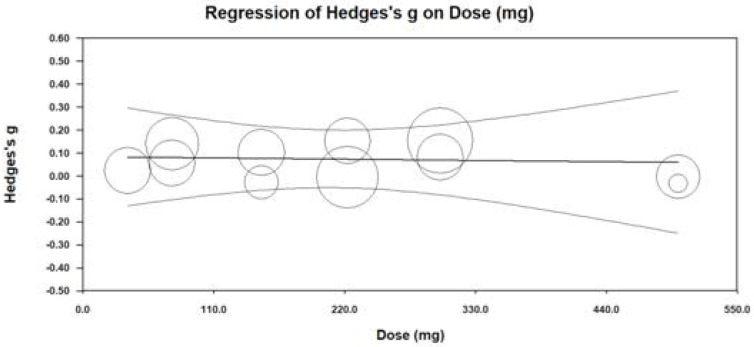
Meta-regression of the association between the guarana dose and the overall study ES. Circles indicate individual studies, with the circle size indicating the relative study weight. Curved lines indicate a 95 percent confidence interval.

**Figure 6:**
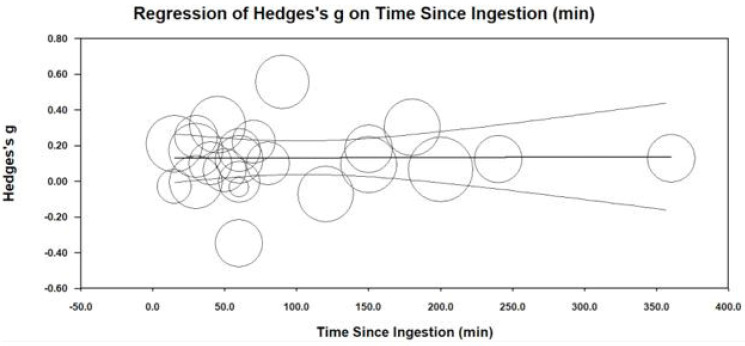
Meta-regression of the association between guarana ingestion time prior to cognitive testing and overall study ES. Circles indicate individual studies, with the circle size representing the individual study weight. Curved lines indicate a 95 percent confidence interval.

Meta-regression analysis was performed for two continuous variables: (1) the dose of guarana and (2) the time following guarana ingestion relative to the study ES. With respect to the dose, the meta-regression showed no significant relationship (R^2^ < 0.001, *p* = 0.9018) (Figure 5). With respect to the time following ingestion, meta-regression again showed no relationship (R^2^ < 0.001, *p* = 0.9541) (Figure 6).

## 4. Discussion

To our knowledge, this is the first systematic review on the effect of guarana on cognitive task performance that is also coupled with a meta-analysis. The main finding of this study is that acute guarana ingestion may influence specific outcomes (e.g., faster response time) in cognitive performance, but this effect is small in magnitude and not congruent with accuracy measures (which were not improved). Moreover, there was no difference when aggregating all of the outcome measures across different cognitive domains for guarana versus a placebo in human studies, despite a trend indicating trivial improvements. There was also no apparent guarana dose–response effect on cognitive performance. This finding was in line with several individual studies [16,22,23]. Several other studies have also demonstrated the split finding that accuracy was not improved despite faster response times during cognitive tasks following guarana ingestion [17,34,35].

One possible explanation for guarana preferentially improving response time in cognitive tasks could be attributed to the caffeine content of guarana. Caffeine acts as an antagonist of adenosine receptors [25] which can increase alertness [14]. Additionally, there is evidence that caffeine improves processing speed [36]. A caffeine dose of 3 mg/kg was reported to decrease response times in a visual search task [37]. In another study, a similar caffeine dose decreased reaction times, but not the accuracy in a choice reaction time task [38]. However, the doses of caffeine in the eight studies in the current meta-analysis were reportedly between (4 to 100 mg), much lower based on absolute dosage. Additional low-dose caffeine studies reported improvements in response time on a range of tasks at 60 [39] and 65 mg [40]. Therefore, it is possible that the caffeine content in guarana could drive the effect on response time. Several of the reviewed studies contained a caffeine comparison trial, with some finding no difference between caffeine and guarana [16,22,34] and others reporting in favor of guarana [35] or caffeine [27]. However, it was not clear if these studies used a matched caffeine dose to the content in the guarana source, making the conclusion regarding caffeine being the causative factor difficult.

Products derived from caffeine-synthesizing plants such as guarana also contain additional bioactive substances which may facilitate mental performance benefits which potentially could exceed those expected from its caffeine content alone [41]. Flavonoids have been linked to improvement in mental processing speeds [26]; however, the available published data are limited. Kennedy [19] used a cocktail with multi-vitamins and minerals added to guarana and found enhanced speed and accuracy of a mentally demanding task (versus the vitamin–mineral complex alone). Only one study [18] in the present meta-analysis utilized multiple doses of guarana (and caffeine) making the dose-response question challenging to address. It is possible these added components within guarana may have an additive or synergistic effect on response time but ascertaining the added benefit above caffeine is not clearly seen in the studies included in the present analysis [16,22,27,34,35], which had a caffeine control trial. A brain imaging study [20] indicated increased activation in several areas following guarana ingestion compared to a placebo, including right pre-central gyrus and left and right superior parietal lobes. A detailed analysis of purported mechanisms for guarana is beyond the scope of this review. Other purported physiological mechanisms of guarana have been debated but at the molecular level including mitochondrial biogenesis and/or anti-inflammatory, chemoprotective effects [42,43]. However, these latter benefits might not be realized following a single acute dose compared to caffeine’s known actions. However, it is possible that chronic supplementation (specifically not included in this analysis) might have an impact after several weeks of guarana, which could potentially address the long-term impact of flavonoids on cognitive performance. However, a recent systematic review and meta-analysis examining the chronic effect of guarana supplementation for several weeks on fatigue in cancer patients [12] did not find a difference from the placebo.

Another possibility for alterations in response time, but not accuracy, could be attributed to potential ceiling effects in some cognitive measures. One study [23] suggested that high initial accuracy in some tasks may result in tasks not being sensitive to a change score after intervention. Therefore, accuracy may be impacted in more difficult tasks, but not easier tasks, particularly in healthy adults. Inclusion of the ES from easier tasks may have diluted the overall effect. Due to the variety of cognitive tasks (primarily in domains related to memory or attention) and the limited number of studies, we were unable to investigate this possibility in our analysis. However, this may explain the high degree of heterogeneity in measures of accuracy.

There are several limitations associated with performing this meta-analysis. Firstly, some published studies did not include sufficient data for ES calculation. Additionally, there were few studies which reported inter-trial correlations in the crossover studies. In order to adjust for the missing correlations, we estimated these based on published test–retest reliability scores from several cognitive tests. An additional sensitivity analysis performed with the lowest observed correlation (0.6) for all outcomes did not change the observed overall result (g = 0.08, *p* = 0.16). Due to the unavailability of correlations, we were unable to perform multivariate meta-analysis for dependent ESs. Additionally, the low total number of studies led to the inability to adequately address some other potential moderator variables. Only a few studies matched the caffeine dose to that contained in guarana [22,35] and there was a wide variety of caffeine contents reported in the guarana supplements used. This makes it difficult to ascertain the effect of guarana beyond a matched dose of caffeine. Publication bias suggests that studies from the “grey literature” (not published in peer-reviewed journals) should also be considered [44]. Thus, we included a published abstract from our laboratory, but we may have missed others despite searching in ProQuest. PEDro scores indicated high quality for most studies, but the total study count was relatively low. 

Our overall conclusions are challenged by the fact that various guarana supplements are available globally, some of which have inconsistent levels of caffeine. In the future, we suggest that the caffeine content of guarana be expressed relative to body mass so that results become comparable in the future. The minimal efficacious dose for cognitive performance remains obscure but likely within the dosage of 1–3 mg/kg body mass. Finally, it should be acknowledged that as additional high-quality studies are published in the future, the present conclusions may be altered.

In conclusion, acute guarana ingestion appears to have a small effect on response time indicating faster performance during a variety of cognitive tasks without affecting accuracy. Whether such performance changes are linked to the caffeine content or other bioavailable substances in guarana is unknown. Future studies might investigate this further through matched-dose comparison trials between caffeine and guarana as well as considering cognitive performance trials following chronic guarana supplementation.

## Data Availability

Not applicable.

## Supplementary Materials

The following supporting information can be downloaded at: https://www.mdpi.com/article/10.3390/nu15020434/s1. Table S1: Summary of Studies. Table S2: PeDRO evaluation of included studies.
