# Supplementary material for: Effect of Guarana (Paullinia cupana) on Cognitive Performance: A Systematic Review and Meta-Analysis
Source: Nutrients. 2023 Jan 14;15(2):434. doi: 10.3390/nu15020434 (PMC9865053; doi:10.3390/nu15020434)
Supplement: Supplementary file 1 [file nutrients-15-00434-s001.zip › nutrients-2132425-Table S1 .pdf]

Table S1: Summary of Studies Assessing Acute Ingestion

Reference	Subjects<br><br>N<br>(#female)<br><br>Age (y)	Guarana Dose<br>(Caffeine content)<br><br>#Other Treatment	Time of Testing After Ingestion (min)	Guarana Source	Cognitive Tests	Parameter Measured	Effects (p<0.05) G vs. P	Effects (p<0.05) of G vs. Other Treatment
Galduróz 1994 * [16]	30(24F)<br><br>R = 24-35	500 mg Capsule (CAF = 2.1%)<br><br>#- CAF (12.5 mg)	60	NR	Digit Span<br>Free Recall<br>Digit Symbol<br>Cancellation	Immediate Memory (SC)<br>Short Term Memory (SC)<br>Attention (SC)<br>Attention (SC)	ND<br>ND<br>ND<br>ND	ND for G vs. CAF- all tasks
Haskell 2007 [18]	26 (NR)<br><br>X = 21.4	37.5, 75, 150, 300 mg Capsule (CAF = 11-12%)	60, 180, 360	PC-102, Pharmaton, SA	Computerized Assessment Battery	Attention (RT)<br>Attention (%)<br>Memory (RT)<br>Secondary Memory (%)<br>Working Memory (%)	ND<br>ND<br>ND<br>+ 37.5mg(MT) 75mg(MT)<br>ND	
Kennedy 2004 [17]	28 (19F)<br><br>X = 21.4	75 mg Capsule (CAF = 11-13%)<br><br>#-G (75mg) +ginseng,<br><br>#-Ginseng	60, 150, 240, 360	Dried Ethanolic Extract	Computerized Assessment Battery<br><br><br><br><br><br>Logical Reasoning<br><br>Sentence Verification<br><br>Serial Threes Subtraction<br><br>Serial Sevens Subtraction	Attention (RT)<br>Attention (%)<br>Memory (RT)<br>Secondary Memory (SC)<br>Working Memory (SC)<br>Reasoning (RT)<br>Reasoning (%)<br>Verification (RT)<br>Verification (%)<br>Attention/Working Memory (SC)<br>Attention/Working Memory (%)<br>Attention/Working Memory (SC)<br>Attention/Working Memory (%)	+ G(60,240,360) GM(150, 240,360)<br>ND<br>+ GM(240, 360)<br>+ G(150)<br>ND<br>ND<br>ND<br>+ G(2.5,4,6) GM(2.5)<br>ND<br>ND<br>+ G(2.5,4) GM(4,6)<br>+ G(1,2.5,4,6) GM(1,6)<br>- G(4) + GM(1,2.5)	
Kennedy 2008 * [19]	130 (70F)<br><br>X = 21.0	222.2 mg in 200 ml water (CAF = 40 mg)	30, 40, 50, 60, 70, 80	Berocca Boost (Vitamins/ Minerals)	Serial Threes Subtraction<br><br>Serial Sevens Subtraction<br><br>Rapid Visual Information Processing	Attention/Working Memory (SC)<br>Attention/Working Memory (%)<br>Attention/Working Memory (SC)<br>Attention/Working Memory (%)<br>Visual Processing (RT)<br>Visual Processing (%)	ND<br>ND<br>ND<br>ND<br>+ (30,40,50,60,80)<br>+ (30,40,50,60,70,80)	

						Visual Processing False Alarms (FA)	ND	
Penna (unpub-lished) [22]	11 (1F)<br><br>X = 20	500 mg Capsule (CAF = 100 mg)<br><br>#-CAF (100 mg)	60, 135	Nutricost Guarana Capsules	Simon Task<br><br>N-Back Task	Response Inhibition (RT)<br>Response Inhibition (%)<br>Working Memory (RT)<br>Working Memory (%)	ND<br>ND<br>ND<br>ND	ND for G vs. CAF -all tasks
Pomportes 2014 [35]	56 (24F)<br><br>X = 28.6	300 mg G in water (CAF = 100 mg)<br><br>#-CAF (100 mg)	15, 30, 45, 60, 90, 120, 150, 180	Isoxan actiflash (Vitamins/ Minerals)	Go/No-go task<br><br>Simple Reaction Time	Response Inhibition (RT)<br>Response Inhibition (%)<br>Reaction Time (RT)		+ Go/No-go RT in G vs CAF at 45-90min
Pomportes 2019 [34]	10 (4F)<br><br>NR	150 mg G in 250 ml water (x2) (CAF = 50 mg (x2))<br><br>#-Carb (6%), CAF (200 mg)	25, 80 (AE)	Isoxan actiflash (Vitamins/ Minerals)	Simon Task	Response Inhibition (RT)<br>Response Inhibition (%)	ND<br>+ (80 (AE))	ND G vs CAF and Carb
Scholey 2013 [20]	20 (12F)<br><br>X = 28.4	222.2 mg G in 330 ml water (CAF = 40 mg)<br><br>#-MVM Complex	30	Berocca Boost (Vitamins/ Minerals)	Serial Threes Subtraction<br><br>Serial Sevens Subtraction<br><br>Rapid Visual Information Processing	Attention/Working Memory (RT)<br>Attention/Working Memory (%)<br>Attention/Working Memory (RT)<br>Attention/Working Memory (%)<br>Visual Processing (RT)<br>Visual Processing (%)	+ (CB)<br>+ (CB)<br>ND<br>ND<br>ND<br>ND	+ Serial Threes RT and % for G vs MVM
Veasey 2015 [21]	40 (0F)<br><br>X = 21.4	222.2 mg G in 250 ml water (CAF = 40 mg)	30, 75, 150	Berocca Boost (Vitamins/ Minerals)	Picture Presentation<br><br>Word Presentation/Recall<br>Choice Reaction Time<br><br>Rapid Visual Information Processing<br><br>Numeric Working Memory	Visual Memory (RT)<br>Visual Memory (%)<br>Immediate Memory (%)<br>Choice (RT)<br>Choice (%)<br>Visual Processing (RT)<br>Visual Processing (%)<br>Working Memory (RT)<br>Working Memory (%)<br>Working Memory (RT)	ND<br>ND<br>ND<br>ND<br>ND<br>ND<br>ND<br>ND<br>+ (CB)<br>ND	

					Delayed Word Recall<br>Word Recognition<br>Picture Recognition	Working Memory (RT)<br>Working Memory (%)<br>Visual Memory (RT)<br>Visual Memory (%)	ND<br>ND<br>+ (CB)<br>ND	

Supplementary Table S1. Summary of Studies Assessing Acute Ingestion of Guarana (n = 9): Description of studies meeting the inclusion criterion when examining effects of acute guarana ingestion on cognitive performance compared to a placebo or control condition (may contain other substances but otherwise no guarana).

Symbols: **+** = Significantly in favor of Guarana (Guarana had faster response time or higher accuracy), **-** = Significantly in favor of placebo X = Mean, ***** = Independent Group Design, Abbreviations: F = Females R = Range, NR = Data Not Reported, G = Guarana Ingestion, P = Placebo, CAF = Caffeine, GM = Guarana Ingestion Mixed with Other Nutrients, MT = Mean of All Time Points, CB = Change From Baseline Condition, AD = All Doses, AE = After Exercise, % = Accuracy, RT = Response Time, FA = False Alarms Generated, SC = Score, ND = No Significant Difference
