# Supplementary material for: Effect of Guarana (Paullinia cupana) on Cognitive Performance: A Systematic Review and Meta-Analysis
Source: Nutrients. 2023 Jan 14;15(2):434. doi: 10.3390/nu15020434 (PMC9865053; doi:10.3390/nu15020434)
Supplement: Supplementary file 1 [file nutrients-15-00434-s001.zip › PEDro Table S2 Final.pdf]

Table S2. PEDro score: Experimental Studies Included in Meta-Analysis.

Criteria	Study							
	**Galduróz 1994 [16]**	**Haskell 2007 [18]**	**Kennedy 2004 [17]**	**Kennedy 2008 [19]**	**Penna (unpublished) [22]**	**Pomportes 2014 [35]**	**Pomportes 2019 [34]**	**Veasey 2015 [21]**
1. Eligibility criteria were specified	YES	YES	YES	YES	YES	YES	YES	YES
2. Subjects were randomly allocated to groups	YES	YES	YES	YES	YES	YES	YES	YES
3. Allocation was concealed	YES	YES	YES	YES	YES	YES	YES	YES
4. The groups were similar at baseline regarding the most important prognostic indicators	YES	YES	YES	YES	YES	YES	NO	YES
5. There was blinding of all subjects	YES	YES	YES	YES	YES	YES	YES	YES
6. There was blinding of all therapists who administered the therapy	YES	YES	YES	YES	YES	YES	NO	YES
7. There was blinding of all assessors who measured at least one key outcome	YES	YES	YES	YES	YES	YES	NO	YES
8. Measures of at least one key outcome were obtained from more than 85% of the subjects initially allocated to groups	YES	YES	YES	YES	YES	YES	NO	YES
9. All subjects for whom outcome measures were available received the treatment or control condition as allocated or, where this was not the case, data for at least one key outcome was analysed by "intention to treat"	YES	YES	YES	YES	YES	YES	YES	YES
10. The results of between-group statistical comparisons are reported for at least one key outcome	YES	YES	YES	YES	YES	YES	YES	YES
11. The study provides both point measures and measures of variability for at least one key outcome	YES	YES	YES	YES	YES	YES	YES	YES
**TOTAL PEDro SCORE**	11/11	11/11	11/11	11/11	11/11	11/11	7/11	11/11

Supplementary Table S2. The PEDro quality scores, for all studies included in the meta-analysis, were ≥ 7. Only one study had a score below the maximum score of 11.
